# Design and Analysis of a Slot Photonic Crystal Waveguide for Highly Sensitive Evanescent Field Absorption Sensing in Fluids

**DOI:** 10.3390/mi11080781

**Published:** 2020-08-15

**Authors:** Reyhaneh Jannesari, Gerald Pühringer, Thomas Grille, Bernhard Jakoby

**Affiliations:** 1 Institute for Microelectronics and Microsensors, Johannes Kepler University, 4040 Linz, Austria; gerald.puehringer@jku.at (G.P.); bernhard.jakoby@jku.at (B.J.); 2 Infineon Technologies Austria AG, 9500 Villach, Austria; thomas.grille@infineon.com

**Keywords:** photonic crystal, slot waveguide, evanescent field absorption

## Abstract

The design and modeling of a highly sensitive sensor based on a slot photonic crystal waveguide (slot-PCWG) is presented. The structure consists of cylindrical air rods drilled in a dielectric slab on a triangular lattice, which are filled with SiO_2_. The waveguide is formed by removing elements from the regular photonic crystal grid in a row, and embedding a slot in the center position. This concept allows for a vast enhancement of the evanescent field ratio, leading to a strong overlap between the field of the waveguide mode and the analyte. In the present work, we show that the sensitivity at the constant slab thickness of the slot-PCWG modes is greatly enhanced, up to a factor of 7.6 compared with the corresponding PCWG modes or Si-slab WGs. The finite-difference time-domain (FDTD) technique and plane wave expansion (PWE) methods were used to study the dispersion and profile of the PCWG mode. The simulation results show the potential of this design, which will be fabricated and tested in the following steps of the project.

## 1. Introduction

Integrated optical sensing technology is expected to play an increasing role in lab-on-a-chip applications, particularly because of their small size [1,2]. Many photonic devices have been proposed as optical sensors, such as micro-ring resonators [3,4,5], slot waveguides [6,7], and photonic crystals [8,9], as well as those based on Bragg gratings [10,11]. However, the type of waveguide that is the most currently used is a slab structure. The sensing is performed by the evanescent tail of the modal field in the cover medium, where the sensitivity is related to the strength of the light–matter interactions [12]. This sensing operation consists of measuring the change in intensity of the mode as a result of absorption in the covering medium. However, many light guiding mechanisms feature only a moderate overlap of the guided optical field with the sample, which results in severe limitations for the maximum sensitivity achievable under specific constraints on the waveguide geometry (i.e., waveguide length, slab thickness, bends, etc.). The achieved measurement sensitivity depends on the optical field distribution in the analyte, so one of the most important design tasks is optimization to maximize its achievable sensitivity concerning the geometric constraints. Photonic crystals (PhCs) are among the most important candidates for improving the performance of slab-based guided-wave devices. They can confine and guide light through narrow channeled waveguides and around very tight bends, with sizes in the order of optical wavelengths. Because of steady improvements in fabrication technology and an increased understanding of the loss mechanisms in these structures, the performance of these structures has seen remarkable development for the past years [13]. At the same time, slot waveguides are able to confine light outside the conventional high-index guiding material. This work presents the concept and design of a new platform, which provides higher sensitivities by combining both concepts to spatially confine the peak energy of the guided mode within the desired sample (analyte). The slotted photonic crystal waveguide (SPCWG) can combine the ability to confine light in the low-index slot with the slow light enhancement available from PCWG [12]. Some preliminary results have been presented in the literature [5]. We compute the dispersion diagrams and the field distributions of these resonances with a plane wave expansion (PWE) method [14]. We then perform finite-difference time-domain (FDTD) simulations [14] to determine, in real time, the interaction between the resonances and material. The fabrication, which is highly CMOS compatible, as well as the experimental verification of the increased sensitivity, are planned on being demonstrated in the near future.

## 2. Optimum Waveguide Design

In this study, as an example, we considered a mid-IR wavelength of λ = 5.85 µm, which is reported to be suitable for the determination of deterioration in lubrication oil [15]. As silicon is transparent in the mid-IR, it is particularly attractive to utilize silicon MEMS technology. Optimized values for the thickness and the length of the waveguide can be found by calculating the maximum change of the normalized power of the guided mode. This allows for defining a sensitivity (figure of merit) using the following equation [16],

$$S=\frac{P_{L}^{aged}-P_{L}^{fresh}}{P_{0}}$$

where $P_{L}^{fresh}$ and $P_{L}^{aged}$ are the power at the output of the waveguide with length L, if the waveguide is loaded either with fresh (n=1.5+0.00128i) or aged oil (n=1.5+0.00184i), respectively.

**Figure 1:**
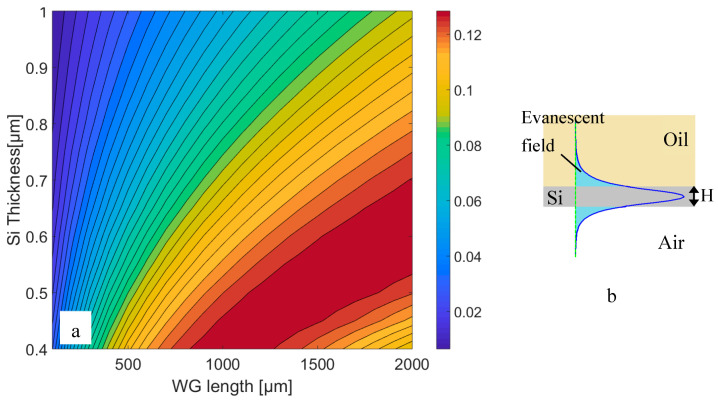
(**a**) Sensitivity of a silicon slab waveguide for different thicknesses as a function of the slab length. (**b**) Schematic view of the electric field distribution in a Si slab with thickness H.

**Figure 2:**
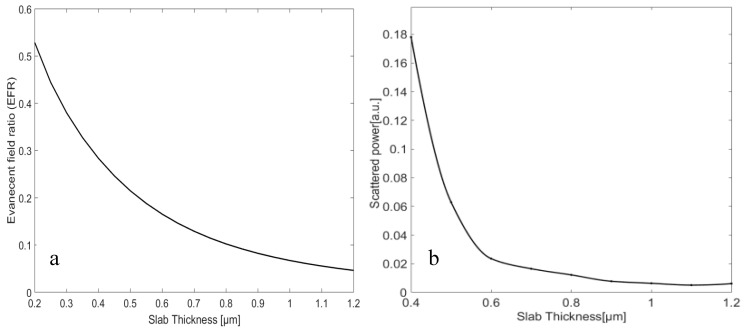
(**a**) Evanescent field ratio (EFR) of the fundamental slab mode versus slab thickness. (**b**) Far-field scattering power of the slab mode versus the slab thickness.

Figure 1 presents the sensitivity of a Si slab (*n* = 3.57) waveguide with different thickness (H) as a function of the slab length (L). A rather good sensitivity can be achieved with either a rather thin slab or a thicker slab with a long length. In this calculation, the scattering loss due to roughness is ignored. Therefore, a short waveguide is more favorable. However, for applications such as coupling to an integrated infrared emitter [17], the utilization of a rather thick slab waveguide would facilitate efficient coupling. Simultaneously, the mode is well confined into the Si-slab in this case, but features a low evanescent field ratio (EFR). The evanescent field ratio, which is a crucial parameter for the sensing application, is defined using the following equation [7]:

$$EFR=\frac{\int_{\mathrm{analyte}}^{}\vec{S}.\vec{n}dydz}{\int_{\mathrm{all}}^{}\vec{S}.\vec{n}dydz}$$

where $\vec{S}$ is the Poynting vector and $\vec{n}$ is the unit normal vector pointing in the direction of propagation. The surface integrals extend over the cross-sectional plane of the slab waveguide. The EFR of the slab WG as a function of the slab thickness is plotted in Figure 2. It can be seen that EFR decreases as the slab thickness increases. The inset of Figure 2 presents the scattered power into the far-field, due to the roughness for a slab waveguide with different thicknesses and $L_{C}/\sigma =20$, where σ is the root mean square (RMS) of the roughness amplitude and $L_{C}$ denotes the roughness correlation length [18]. As can be seen, increasing the slab thickness reduces the effect of the total scattering of roughness, because the slab mode is more confined into the slab, and the intensity of the electric field on the slab surface is less for the thicker slabs. Therefore, conventional methods (i.e., slab WG) of evanescence-field absorption demand rather thin slabs in order to achieve the required EFRs and, subsequently, sensitivities. However, the requirement of a thin slab stands in sharp conflict with the benefits of a thicker slab (i.e., better source coupling and less spurious leakage of the WG mode due to scattering loss). This area of conflict can be resolved with PCWGs.

## 3. Photonic Crystal Slabs

**Figure 3:**
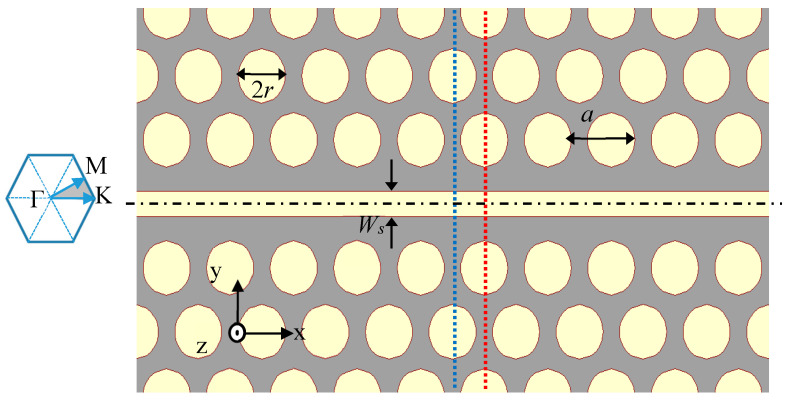
Two-dimensional schematic view of the slot-photonic crystals (PC) waveguide. The blue and red dashed lines define the position of the monitors used to calculate the evanescent field ratio (EFR). The inset shows the high-symmetry points at the corners of the irreducible Brillouin zone (shaded gray).

Photonic crystal (PC) slabs are known because of their potential ability to three-dimensionally control light propagation. The photonic band structure controls propagation in the crystal plane, and the index guiding capabilities confine light in a vertical direction [13]. The most common use of the PC slabs consists of periodic arrays of holes in dielectric slabs. The ability of PCs to control the flow of light, confine light in a small volume, and enhance light–matter interactions makes them promising candidates for sensor applications [3]. By employing photonic crystals, one can engineer the dispersion properties of the guided modes. This enables designs where electromagnetic radiation strongly interacts with an analyte at a specific wavelength, λ, determined by the periodicity and the geometric design of the photonic crystal [19]. Another feature of PCs is the ability to create local states in the bandgap by locally creating defects in the periodic lattice (crystal defects) [20]. A series of defects in photonic crystals will break the existing symmetry of the photonic crystal, and a narrow defect mode is created inside the photonic bandgap, which is a waveguide mode. The corresponding guided mode is confined to these defects, and light can only travel along the waveguide [21]. The basic structure in this work is a hexagonal lattice of air holes introduced into a silicon slab (*n* = 3.57) with period *a*. The holes, with a radius r=0.37a, are filled with SiO_2_ (*n* = 1.279). Here, the WG is created by removing one line of raw of holes in the ΓK direction. Figure 3 shows a schematic view of the PC waveguide with an additional slot within the waveguide.

**Figure 4:**
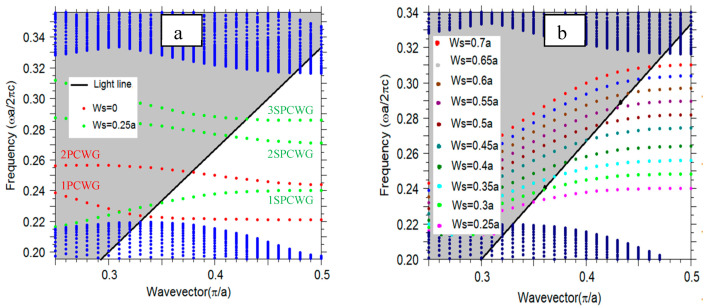
(**a**) The blue dots present the dispersion band diagram of a 2D triangular lattice of holes with a radius of r=0.37a in Si with TE polarization along the ΓK direction (shown in the inset of Figure 3). The modes above the light line (black line), the shadowed gray area, are the radiating modes. The red dots present a band diagram of the PC waveguide (without slot), the green dots present a band diagram of the slot PC waveguide with a slot width of $W_{s}=0.3a$. (**b**) Dispersion band diagram of the first slot PCWG (1SPCWG) for different slot widths.

**Figure 5:**
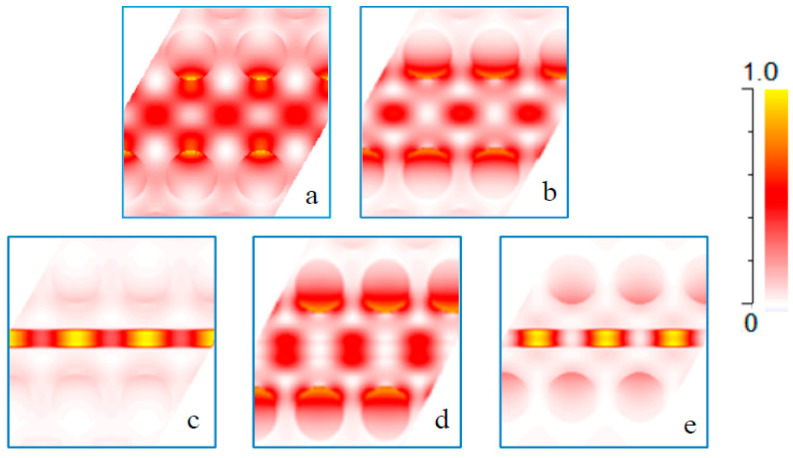
Spatial distribution of the electric fields for the (**a**) first photonic crystal waveguide (PCWG) mode, (**b**) second PCWG mode, (**c**) first slot PCWG mode, (**d**) second slot PCWG mode, (**e**) third slot PCWG at the K=0.5π/a point. The white color represents low intensity and the red color represents high intensity.

Figure 4a shows the dispersion diagram of the TE PC mode, which has the electric field dominantly oriented in the photonic crystal plane. The blue dots show the dispersion bands of a 2D photonic crystal with a bandgap that ranges from 0.211a/λ−0.305a/λ. Only modes below the light line (black line in Figure 4) can be confined into the photonic crystal slab. The modes in the gray shadow area are the radiating modes. As mentioned above, a photonic crystal waveguide is formed by removing one line of SiO_2_ holes, creating a series of defects. The photonic crystal waveguide (PCWG) creates additional bands in the bandgap of the unperturbed crystal. The red dots present the two PCWG modes. The WG modes are laterally confined to the WG area. Therefore, they cannot propagate into the PC. These two modes can be classified using their symmetry, as follows: The lower energy mode (1PCWG in Figure 4a) is an even mode with respect to the reflection plane at the centered midway of the PCWG (shown with dash-dot line in Figure 3), in which the H-field profile does not change its sign upon reflection in this plane. The higher energy mode (2PCWG in Figure 4a) is an odd mode in which the H-field changes sign upon reflection in the symmetry plane. For both symmetries, the E-field is mostly localized in high dielectric regions (Figure 5a,b). In this work, we are interested in determining a mode to enhance the light–matter interaction of a photonic crystal WG mode by a fluid analyte in a rather thick slab. In such a way, one can also facilitate the efficient coupling of polarized light from an integrated infrared emitter [17]. The photonic crystal waveguide mode features a high confinement of the electric field inside the Si slab. The proposed structure is a slot photonic crystal waveguide, in which the guided mode is an eigenmode of the photonic crystal and therefore, fundamentally, has a low loss. The optical field in this structure is enhanced and confined in the low index material, which is a typical feature and strength of slot WGs. Hence, the designed device has a rather high evanescent field ratio, resulting in a strong light–matter interaction, which contributes to a high sensitivity. Only TE polarized light is considered here. A slot with width $W_{s}$ is embedded in the center position of the waveguide. The slot is also filled with SiO_2_ (shown in Figure 3).

Introducing a slot into the PCWG pushes the WG modes into higher energies. In addition, an extra mode detached from the bulk PC mode merges into the band-gap. The H-field profile of this mode has an even symmetry. The green dots in Figure 4a show the slot photonic crystal waveguide (SPCWG) modes. Changing the slot width allows for tuning the energy of the SPCWG mode and the evanescent field ratio. Changing the distance between the slot edge and the first row of holes can also tune the energy of the SPCWG mode. Increasing the distance shifts the SPCWG modes to lower energies, but these are out of the scope of the present work). Increasing the slot width shifts all SPCWG modes to higher energies, however the second SPCWG mode moves at a faster rate than the third SPCWG mode into the higher energies. For our PC configuration at a slot width of $W_{s}=0.14a$, the second SPCWG crosses the third SPCWG mode. Therefore, the symmetry of the SPCWG modes for $W_{s}>0.14a$ is even, odd, and even for the first SPCWG, second SPCWG, and third SPCWG (Figure 4a), respectively. Figure 5c,d presents, as an example, the E-field profile of the SPCWG modes for $W_{s}=0.34a$ at K=0.5π/a. The two modes with even symmetry have an electric field localized in the slot regions, which is useful for the application of this work. Our goal is to find a mode featuring a high fraction of electric field in the area above the SiO_2_ slot. Choosing small values for *W_s_* (e.g., 0.25*a*) results in a significant field enhancement above the slot, but two unwanted WG modes exist within the bandgap, as can be seen in the dispersion relation in Figure 4a. Increasing *W_s_* above a value of 0.5*a* drifts modes to higher energies, and therefore pushes the parasitic modes out of the gap, leading to a single SPCWG mode in the gap. Figure 4b presents the dispersion diagram of the first SPCWG mode, which crosses the bandgap when increasing the slot widths. Additionally, the flattening of the dispersion (see Figure 4b) implies a lower group velocity, which results into a higher sensitivity. Thus, the sensitivity can be optimized by choosing a large value of *W_s_*, while maintain a confined guided mode and a feasible propagation length for the WG. In particular, the mode at K=0.5π/a with normalized energy, ωa/2πc=0.281, entails an excellent mode separation by the adjacent bandgaps and a maximum light–matter interaction due to the low group velocity (small slope near the edge of the Brilouin zone). The plane wave expansion (PWE) method was used to calculate the photonic bandgap and propagating modes of the structure.

## 4. Result and Discussion

Many of photonic sensors, such as a rib or strip waveguide, utilize only the evanescent tail of the optical mode for detecting, which limits their sensitivity. Huang et al. [22] reported three kinds of waveguides (strip, rib, and slot) on a silicon-on-sapphire platform at a mid-IR for gas sensing, based on the evanescent field absorption. In their work, the slot waveguide provides the highest EFR (25%) for a slot height of 0.4µm. Siebert et al. achieved a 25% EFR for a free-standing rib waveguide [23]. In the literature [24], a numerical investigation of the slot waveguides for CO_2_ sensing using a polysilicon platform was presented, and an evanescent field ratio as high as 42% for a slot waveguide was reported. The small footprint of the above-mentioned devices presents opportunities for dense arrays of sensing elements and a lab-on-chip device. However, they suffer from a lack of integrated light source and WG coupling [1,17,25]. This situation might be improved by increasing the WG height, as various integrated coupling devices (taper, resonant emitters, etc.) benefit from increased slab heights. On the other hand, there is a trade-off between the EFR and the height of the waveguide, stating that reducing the height increases sensitivity [22]. This is a key limiting factor of many devices in the literature. To overcome the limitation of the thin slab, we performed studies on a slot photonic crystal waveguide based on a rather thick polysilicon slab with a high EFR. Slotted photonic crystals have been developed towards a realistic and promising platform for lab-on-a-chip sensors. The great advantage over many other photonic devices is the strong confinement of light in a low index material, not only spatially, but also temporally, because of a combination of slot waveguide and photonic crystal effects [12]. This strong confinement of light in the low index region is the key advantage of the slotted photonic crystal, as it promotes strong light–matter interactions between the slot mode and the analyte.

**Figure 6:**
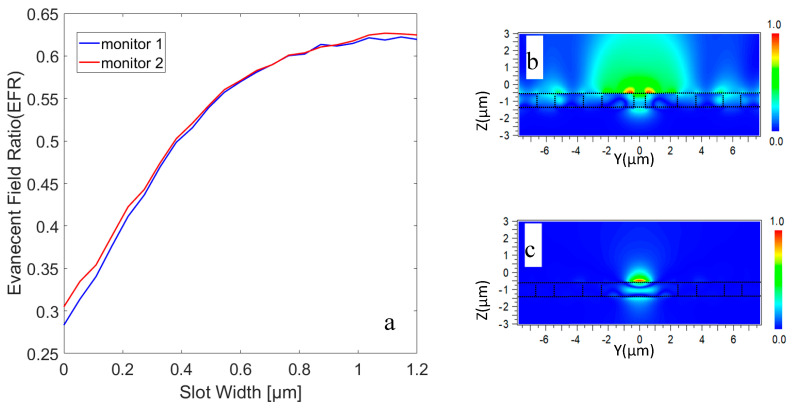
(**a**) EFR versus slot width for the slot PC waveguide in two different cross-sections featuring high symmetry in the PC lattice. The position associated with the blue and the red lines is indicated in Figure 5. (**b**) The profile of the Poynting vector in x direction of a quasi-TE mode in a waveguide with $W_{s}$ = 0.9 µm and (**c**) $W_{s}=0$ µm.

Hereafter, we consider a three-dimensional (3D) slot waveguide structure with a finite height. In this case, the modes have quasi-TE polarization, which means the major E-field component is along the y direction. The quasi-TE mode of the 3D slot waveguide structure was calculated with a full-vectorial FDTD with a non-uniform grid mesh. We assumed a free-standing slab waveguide sandwiched between a cladding cover (oil, *n* = 1.5) and a substrate (air, *n* = 1), and the rectangular cross-section of the slot, with a height of H=0.9 µm, is filled with SiO_2_. As already mentioned, a thicker slab height is preferable for better source coupling. The EFR of the slot versus the slot width for the SPCWGs in two different cross-sections featuring high symmetry in the PC lattice (shown in Figure 3 with dotted blue and red line) was calculated. The evanescent field ratio can be pushed to about 63% (see Figure 6). This value is already up to a factor of 7.6 larger than the evanescent field ratio of the associated slab WG (slab height = 0.9 µm). The simulated electric field profile is given in terms of the field component Ey of the SPCWG modes, with $W_{s}=0.9$ µm, as shown Figure 6b. Figure 6c shows the profile of the transverse electric field of a quasi-TE mode in a gapless waveguide (*Ws* = 0).

## 5. Conclusions and Future Directions

In conclusion, we presented a design for a slot photonic crystal waveguide facilitating the enhancement of the interaction between the analyte and light, which leads to the enhancement of the effectively detected analyte absorption. The symmetry of the photonic crystal waveguide modes with and without a slot was investigated, and an enhancement factor of 7.6 was found in the evanescent field ratio compared with a slab waveguide with a similar thickness was achieved by tuning the slot width. We demonstrated the potential for highly sensitive liquid detection with a slot photonic crystal waveguide. This high sensitivity could be achieved because of the large overlap of the waveguide mode with the analyte. As mentioned before, the experimental validation of the performance of the designs presented in this work is the next goal of this project. The fabrication techniques can easily be adapted from CMOS technology.
